# Inactivation of the CovR/S Virulence Regulator Impairs Infection in an Improved Murine Model of *Streptococcus pyogenes* Naso-Pharyngeal Infection

**DOI:** 10.1371/journal.pone.0061655

**Published:** 2013-04-25

**Authors:** Faraz M. Alam, Claire E. Turner, Ken Smith, Siouxsie Wiles, Shiranee Sriskandan

**Affiliations:** 1 Section of Infectious Diseases and Immunity, Department of Medicine, Imperial College London, London, United Kingdom; 2 Department of Pathology and Infectious Diseases, Royal Veterinary College, Hertfordshire, United Kingdom; 3 Department of Molecular Medicine and Pathology, University of Auckland, Auckland, New Zealand; Columbia University, United States of America

## Abstract

*Streptococcus pyogenes* is a leading cause of pharyngeal infection, with an estimated 616 million cases per year. The human nasopharynx represents the major reservoir for all *S. pyogenes* infection, including severe invasive disease. To investigate bacterial and host factors that influence *S. pyogenes* infection, we have devised an improved murine model of nasopharyngeal colonization, with an optimized dosing volume to avoid fulminant infections and a sensitive host strain. In addition we have utilized a refined technique for longitudinal monitoring of bacterial burden that is non-invasive thereby reducing the numbers of animals required. The model was used to demonstrate that the two component regulatory system, CovR/S, is required for optimum infection and transmission from the nasopharynx. There is a fitness cost conferred by *covR/S* mutation that is specific to the nasopharynx. This may explain why *S. pyogenes* with altered *covR/S* have not become prevalent in community infections despite possessing a selective advantage in invasive infection.

## Introduction

*Streptococcus pyogenes* is estimated to cause 616 million cases of pharyngeal infection per year, and 663,000 cases of invasive disease [1]. As the human nasopharynx represents the major reservoir for all types of *S. pyogenes* infection, it is essential to develop a better understanding of the factors that influence upper respiratory tract infection.

Despite their limitations, mice play an important role in infectious diseases research [2]. The mouse nasopharynx has structural similarities to the nasal turbinate system in humans [3], although mice lack tonsils [4]. Instead, mice possess nasal associated lymphoid tissue (NALT), which shares some similarity to the tonsils [5] and has been shown to be a target for infection by *S. pyogenes* [6]. Indeed, mice have been used by several groups to investigate *S. pyogenes* in the upper respiratory tract, although there is no consensus on which is the most appropriate strain, sex or age of animal to use [6], [7], [8], [9], [10]. Furthermore, the maximum dose volume posited for establishing infection by previous studies ranges from 5 µl, as determined by administration of colored dye [8], [11], 10 µl as determined by radioactive microspheres [10], [12], to 20 µl volumes [6]. This is an important consideration, as aspiration of the bacteria into the lungs has the potential to trigger a more invasive disease and systemic infection.

It is known that phenotypic differences can exist between nasopharyngeal and invasive *S. pyogenes* isolates, and these have been ascribed to altered activity or mutation of the streptococcal two component regulatory system, *covR/S* [13]. As a result of signalling from the sensor kinase, CovS, CovR represses a range of virulence factors concerned with resistance to phagocytosis, such as the capsule synthesis operon *has*ABC, the DNase, *sda*, and the CXC chemokine protease, SpyCEP [13], [14], [15], [16], [17]. Mutations in *covR/S* de-repress these virulence genes, conferring a selective advantage to *S. pyogenes* in mouse models of invasive infection, leading to greater mortality [15], [18]. However, the impact of such mutations on nasopharyngeal infection is unclear. Isolates of *S. pyogenes* with *covS* mutations bind less well to skin cells in vitro and in vivo than those without the mutation [19]. Furthermore, *S. pyogenes* with mutations in *covS* lack competitiveness in the saliva relative to wild type [20].

In this work, we set out to produce a longitudinally monitored murine model of nasopharyngeal infection, by examining the effect of mouse strain, age and sex on *S. pyogenes* carriage. We evaluated *S. pyogenes* pharyngitis isolates from patients rather than a previously-described mouse-pathogenic strain that lacks a functional copy of the multigene activator, *mga* [21], [22]. The improved nasopharyngeal infection model was used to evaluate the impact of the *S. pyogenes* CovR/S two component regulatory system on longevity and transmission of *S. pyogenes* upper respiratory tract infection.

## Methods

### Ethics Statement

In vivo experiments were performed in accordance with the Animals (scientific Procedures) Act 1986, subject to protocols set out in PPL 70/7379 that were approved by the Imperial College Ethical Review Process (ERP) panel and the UK Home Office.

### Bacterial Strains

The bacterial strains used in this study are given in Table 1 [16], [23], [24]. Streptococcal strains were cultured in Todd Hewitt Yeast broth (THY) or on Columbia Blood Agar (CBA), while Luria Bertani (LB) medium was used for culturing *C. rodentium* ICC180. All strains were grown at 37°C. For animal experiments, *S. pyogenes* was grown without shaking with 5% CO_2_ overnight, centrifuged at 1864×g, (Sorvall RTH 750 Rotor), washed twice in phosphate buffered saline (PBS), and re-suspended in PBS to produce an inoculum of 1–7×10^8^ colony forming units (cfu) per 5 µl. Numbers of viable bacteria within the inoculum were retrospectively assessed by plating of 10^−6^–10^−8^ dilutions of the inoculum onto agar.

**Table 1:** Bacterial strains used in this study.

Strain Designation	Emm type	Species	Description	Ref.
H305	*emm1*	*S. pyogenes*	Scarlet fever reference strain	[23]
H343	*emm2*	*S. pyogenes*	Pharyngitis isolate	[16]
H292	*emm81*	*S. pyogenes*	Blood isolate	[16]
H347	*emm75*	*S. pyogenes*	Pharyngitis isolate	[16]
H494	*emm75*	*S. pyogenes*	Strain H347 with an inactivated *covR/S* operon	[16]
ICC180		*C. rodentium*	Bioluminescent derivative of ICC169	[24]

### Animals

Male and female 5–10 week old CD1, C57BL/6, A/J, BALB/c, FVB/n specific-pathogen free mice (Harlan, UK) were maintained in individually HEPA filtered cages with sterile bedding and free access to sterilized food and water. GLP Mini Fun Tunnels (Lillico), or Des. Res. Mini Mouse Houses (Lillico) were provided in each cage for environmental enrichment.

### Intranasal Infection

Pilot experiments were conducted using ∼10^9^ cfu in doses of 2.5 µl–20 µl bioluminescent *C. rodentium* ICC180 [24] to determine the correct dosing strategy to deliver bacteria to the murine nasopharynx without lung involvement. Bioluminescence (as photons s^−1^ cm^−2^ steridian [sr] ^−1^) from living animals was performed as previously described [24] using an IVIS® 100 system (Perkin Elmer).

For streptococcal infection of the nasopharynx, 1–7×10^8 ^cfu of *S. pyogenes* was administered intranasally using a pipette to mice in a volume of 2.5 µl per nostril under 2–5% isoflurane anaesthesia. Mice were weighed daily; reduction by 20% of original weight was a defined humane endpoint.

### Intramuscular Infection

6×10^8 ^cfu *S. pyogenes* were administered to mice under isoflurane anaesthesia via injection with a 27 gauge needle into the right lateral thigh. Numbers of viable bacteria within the inoculum were assessed by retrospective plating of 10^−6^–10^−8^ dilutions. At 72 hours, the right thigh muscle and ipsilateral inguinal node were extracted, weighed and homogenized into PBS and then plated out onto CBA for bacterial enumeration.

### Nasal Sampling

The level of shedding of *S. pyogenes* from the nasopharynx was assessed longitudinally using direct nasal sampling. The nares of each mouse were gently pressed onto the surface of a CBA plate ten times. Exhaled particulates were streaked out, and the plates were then incubated overnight at 37°C with 5% CO_2_ for bacterial enumeration. In preliminary experiments using naive, non-infected mice, α-hemolytic streptococci, staphylococci, pseudomonads and corynebacteria were recovered, but no β hemolytic Group A streptococci were found to naturally colonize the mouse nasopharyngeal tract.

For experiments where mice had been infected with *S. pyogenes*, β-hemolytic colonies were counted for each mouse and confirmed as *S. pyogenes* through Gram staining, catalase testing, oxidase testing, and Lancefield grouping. No other β-haemolytic bacteria were recovered from the mouse nasopharynx. Kaplan-Meier plots were created to analyse the duration of *S. pyogenes* shedding. Nasal samples were taken for 21 days post inoculation. Mice were determined to have stopped shedding upon the first instance of a nasal sample turning up negative.

In some studies, the nasopharynx was dissected and removed at fixed time points for microbiological culture. To ensure complete extraction of the nasopharynx, the skin and mandibles were removed to expose the cranium. This was then sectioned along the coronal plane at the bregma. The brain tissue within the cranium anterior to this incision was removed, exposing the posterior aspect of the nasal cavity, known as the cribriform plate. The orbits were removed from this tissue via sagittal incisions lateral to the pre-maxilla. The remaining tissue comprised the entire nasal cavity, and NALT. This was homogenized into PBS and serial dilutions plated onto CBA to quantify *S. pyogenes* from the whole nasopharynx.

### Settle Plates

To detect the presence of airborne bacteria within cages of infected mice, CBA plates were placed in the upper rack of the individually HEPA filtered cages (n = 4 plates per cage) and exposed for defined time periods throughout each experiment. Plates then incubated overnight at 37°C and the numbers of *S. pyogenes* colonies, (identified by Gram staining, catalase testing, and Lancefield grouping) were enumerated.

### Histopathology

The head of each mouse was removed at the atlanto-occipital joint and sagittally hemi-sected. One half was fixed in formalin and processed routinely to paraffin wax, while the other was homogenised and plated to assess *S. pyogenes* numbers in the nasopharynx. Paraffin sections were cut at 6 µm and stained with Haematoxylin and Eosin and Gram stains. The degree of nasal damage and inflammation was scored as : No significant abnormality (Intact nasal mucosa and absence of inflammation), Mild (Focal erosion of the mucosa with local neutrophil exocytosis across the affected epithelium), Moderate (Focal necrosis and ulceration of the mucosa with local neutrophilic exocytosis and surface neutrophilic exudation), Marked (Extensive necrosis and ulceration of the mucosa with widespread neutrophilic exocytosis and surface neutrophilic exudation) and Severe (Extensive necrosis and ulceration of the mucosa with widespread neutrophilic exocytosis and surface neutrophilic exudation and with extension of necrosis into underlying stroma). Slides were reviewed and scored by an experienced histopathologist (KS).

### Statistics

For statistical analysis of Kaplan-Meier curves, the Mantel-Cox Logrank test was applied. For statistical analysis of colony count comparisons, a non-parametric Kruskal-Wallis test and Dunn’s post-test were used. *P* values less than 0.05 were defined as significant. Statistics were performed using Prism Graphpad version 5.02. Data are presented as median, ± interquartile range.

## Results

### Volume of Inoculum Determines Distribution within the Respiratory Tract

**Figure 1:**
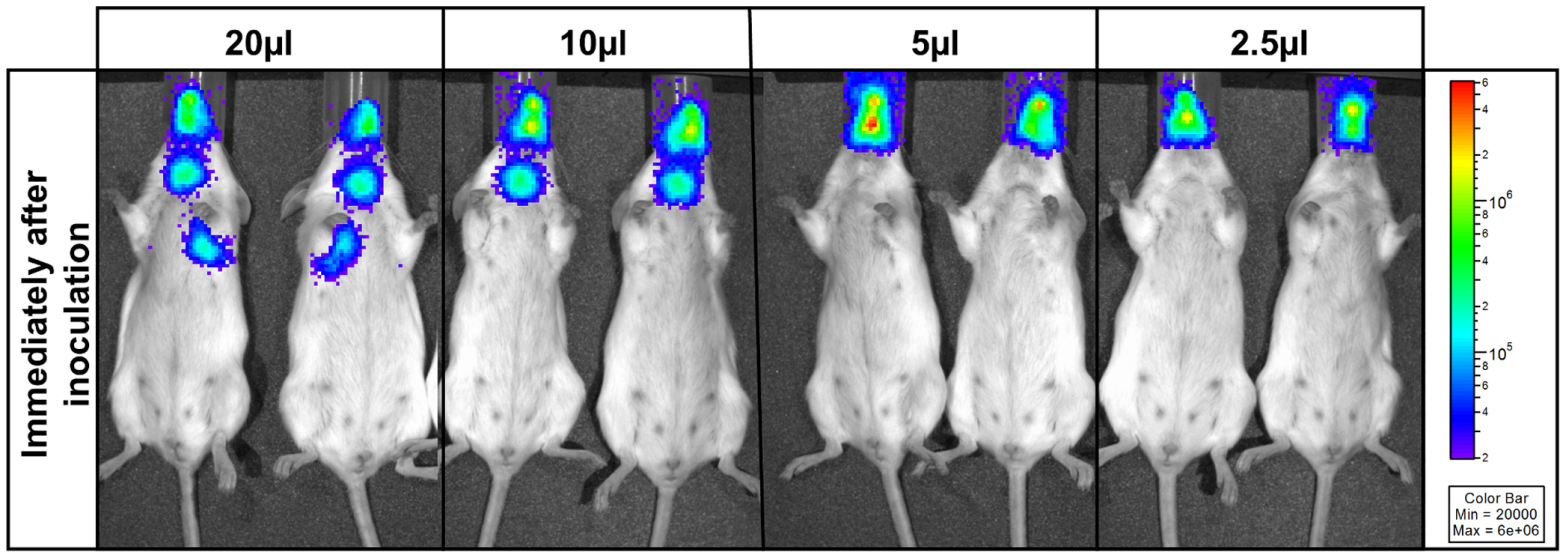
Bioluminescence imaging of bacterial distribution after intranasal inoculation with bioluminescent *C.*rodentium. 10^9^ colony forming units (cfu) of bioluminescent *C. rodentium* were administered intranasally to 8 week old CD1 outbred female mice in 20 µl (n = 3), 10 µl (n = 2), 5 µl (n = 3) and 2.5 µl (n = 2) of PBS. Images were acquired using an IVIS spectrum system, and are displayed as images of peak bioluminescence, with variations in colour representing light intensity at a given location. Red represents the most intense light emission, while blue corresponds to the weakest signal. The colour bar indicates relative signal intensity (as photons s^−1^ cm^2^ sr^−1^). Two representative mice shown for each group.

Bioluminescence imaging demonstrated that dose volumes of 20 µl volume delivered bacteria to the lungs, whereas this was not shown with lower volumes (Figure 1). Any dose volume above 10 µl was deposited in the trachea. Dose volumes of 5 µl and 2 µl did not distribute bacteria to the lungs. As the optimal dose volume for nasopharyngeal deposition without lung involvement or significant nasal clearance was 5 µl (2.5 µl per nostril), this was the volume used in subsequent experiments.

### Nasopharyngeal Shedding as a Method to Longitudinally Monitor S. pyogenes Infection in the Upper Respiratory Tract

**Figure 2:**
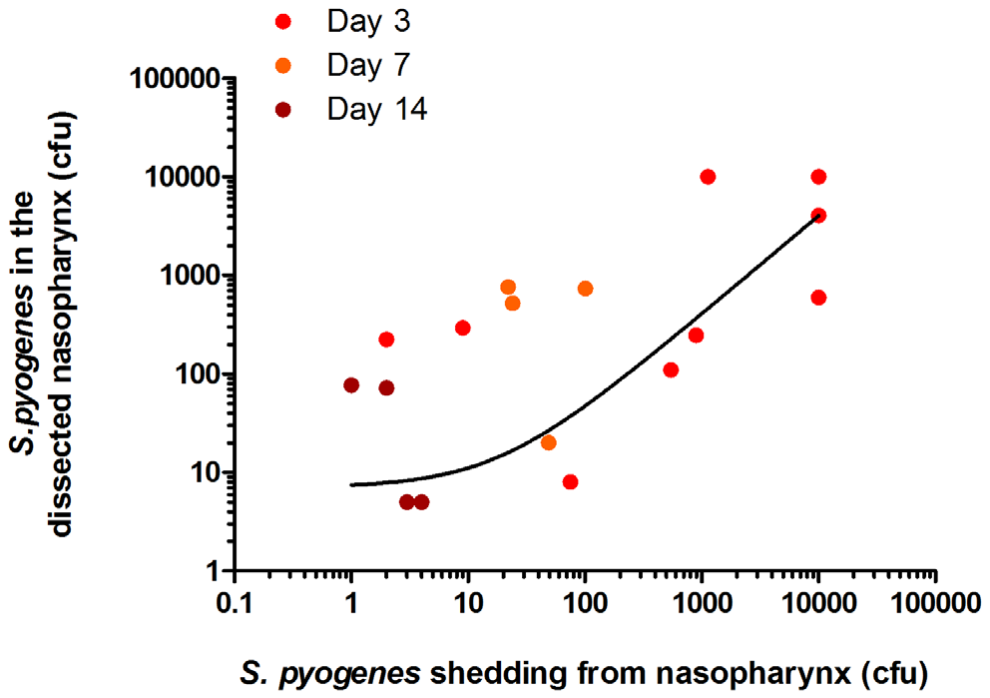
Correlation between *S.* pyogenes recovered from nasal shedding and from nasopharyngeal dissection. Data are pooled from direct nasal samples taken on days 3, 7, and 14 from five week old female FVB/n mice during intranasal infection with *emm75 S. pyogenes* (1.3×10^8^ cfu) were compared to bacterial numbers obtained on dissection on the same days. (r^2^>0.95, n = 36). Data shown from individual mice.

To select an appropriate bacterial strain for development of the model, BALB/c mice were intranasally inoculated with four clinical *S. pyogenes* strains of different *emm* genotypes. An *emm*75 pharyngitis strain was found to have shed for the longest period using direct nasal sampling (Table 2) and was used in subsequent experiments. Importantly, a significant correlation was found between the numbers of colonies recovered from nasal shedding of *S. pyogenes* and bacterial numbers from dissected and homogenised nasal tissue on the same day (Figure 2, r^2^>0.95, n = 36). This longitudinal method of monitoring was therefore employed in subsequent experiments consistent with the principles of the 3Rs (Replacement, Refinement, and Reduction) by reducing the numbers of animals used.

**Table 2:** Duration of shedding of different strains of *S. pyogenes* (7×10^8^ cfu per 5 µl dose) in 8 week old male BALB/c mice after intranasal infection.

	Number of mice shedding *S. pyogenes*		
	Day 1	Day 2	Day 3
***emm*** **1**	0/7	0/7	0/7
***emm*** **2**	1/7	1/7	1/7
***emm75***	4/7	3/7	2/7
***emm*** **81**	1/7	0/7	0/7

### Longevity of Nasopharyngeal Shedding is Greater in FVB/n Mice

**Figure 3:**
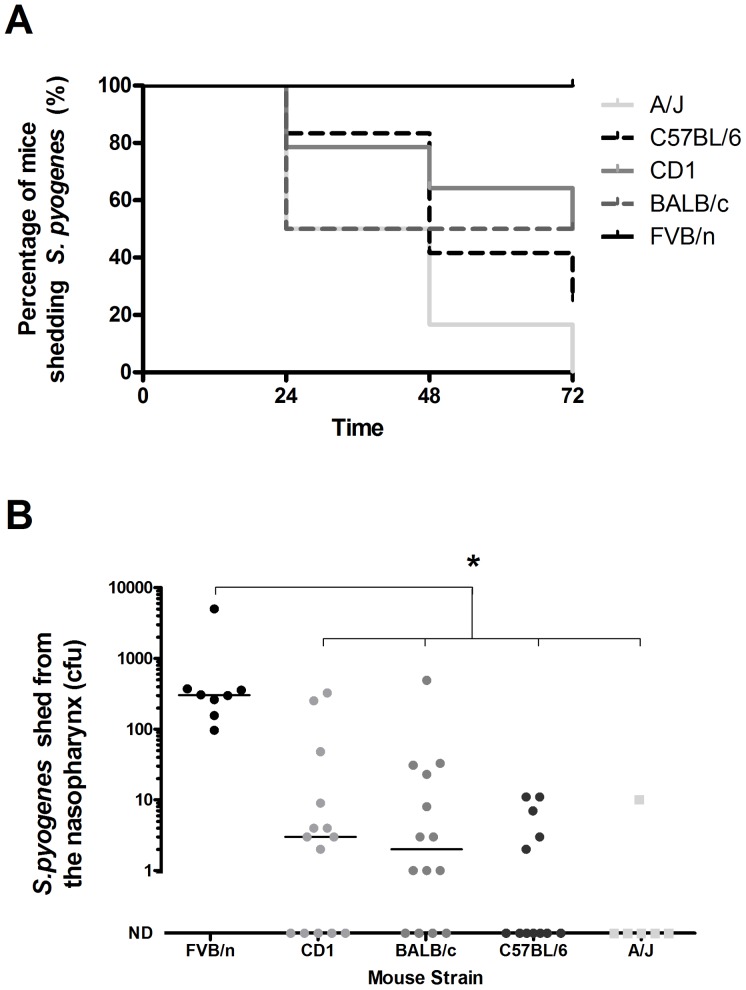
Comparison of nasopharyngeal shedding of *S.* *pyogenes* between different mouse strains. Five week old male mice of different strain backgrounds were inoculated intranasally with *emm*75 *S. pyogenes* (1.5×10^8^ cfu). Duration and quantity of shedding were determined from colonies recovered from direct nasal sampling of the mouse nasopharynx. (A) Kaplan-Meier plot showing percentage of mice shedding *S. pyogenes* in each group. (B) Bacterial counts shed by FVB/n (n = 8) CD1 (n = 14), BALB/c (n = 14), C57BL/6 (n = 12) and A/J (n = 6) at 72 hours. Individual points represent individual mice. (Kruskal Wallis with Dunns Post Test *p*<0.05). Bars indicate the median, ND = no detectable bacteria.

To determine the most appropriate mouse strain for model development, male mice of different host backgrounds were infected intranasally with *emm*75 *S. pyogenes*, and observed longitudinally for 72 h using direct nasal sampling. Data were used to create Kaplan Meier plots to analyse the duration of shedding for each strain of mouse (Figure 3 A). FVB/n mice carried *S. pyogenes* longer and shed significantly more *S. pyogenes* on the final day of the time course than all other strains tested (Figure 3 B). FVB/n mice carried *S. pyogenes* were therefore used for all future studies.

### Gender has an Effect on the Nasopharyngeal Carriage of *S. pyogenes* that is Dependent on the Age of the Mice

**Figure 4:**
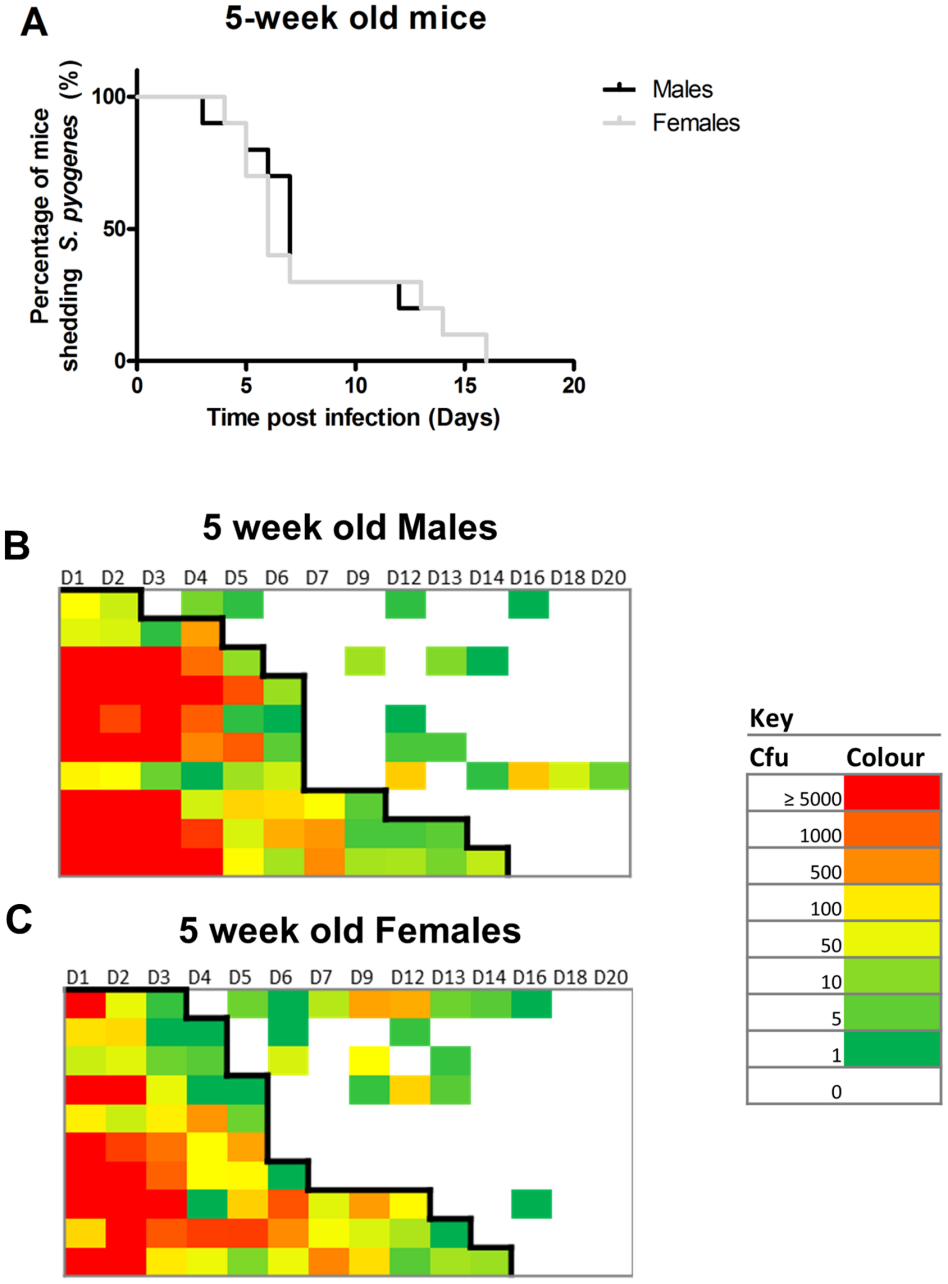
Sex differences in ***S*** **.** **** ***pyogenes*** **nasopharyngeal carriage and shedding intensity in 5 week old mice.** **** Male and female FVB/n mice 5 weeks (A, *p*>0.05 Mantel–Cox Logrank test, n = 10) of age were infected intranasally with *emm75 S. pyogenes* (1.1×10^8^ cfu) and sampled non-invasively through direct nasal sampling over 21 days. Shedding intensity maps display the data from direct nasal samples throughout the time course from the male mice (B) and the female mice (C). Rows indicate individual mice throughout the time course, colours indicate the numbers of *S. pyogenes* recovered from direct nasal samples with red indicating the highest recorded levels of carriage (≥5000 cfu) and green indicates the lowest levels of carriage (1 cfu) and blank blocks indicate no recovery of *S. pyogenes*. Black line indicates survival based on the first loss of carriage.

**Figure 5:**
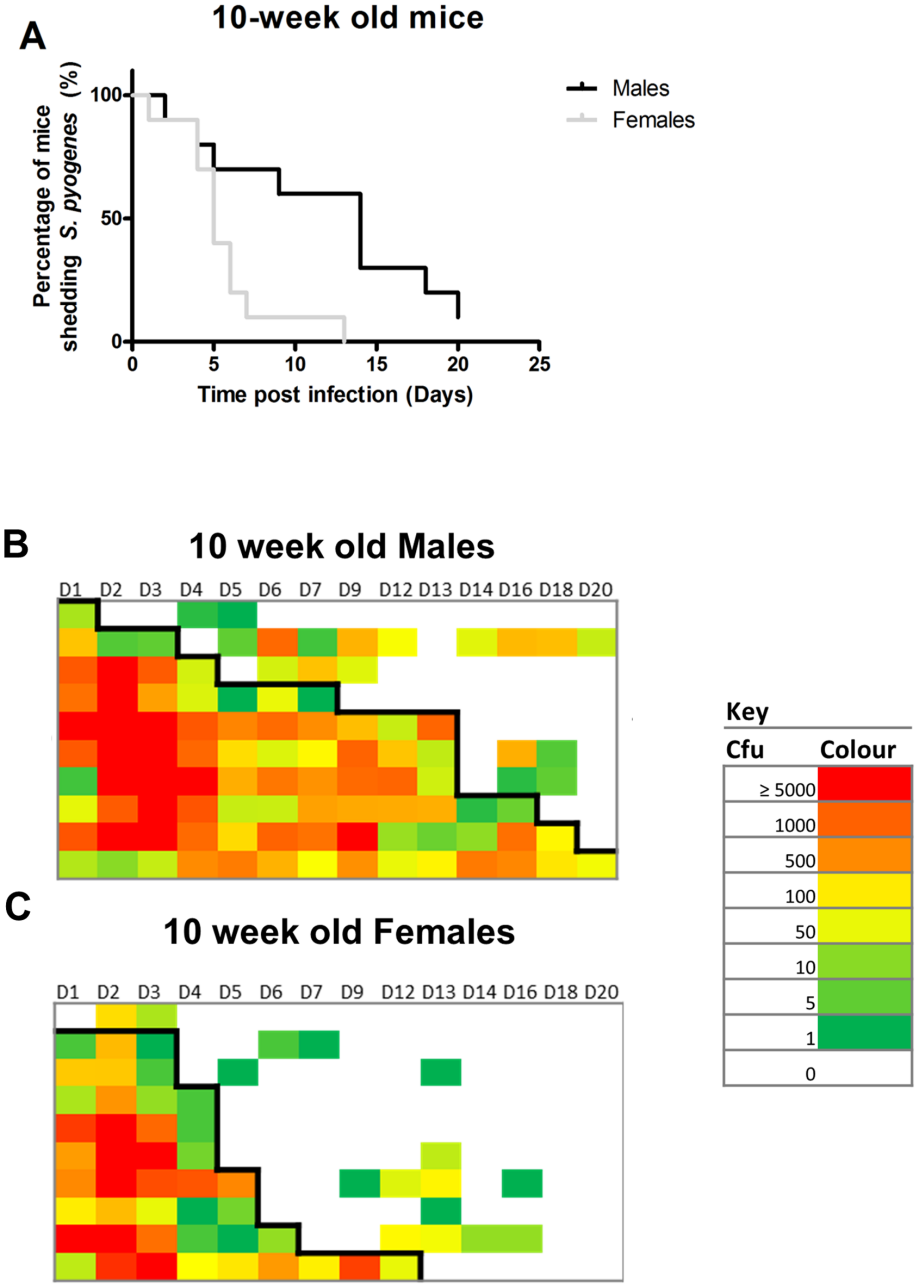
Sex differences in *S*. ***pyogenes*** **nasopharyngeal carriage and shedding intensity in 10 week old mice.** **** Male and female FVB/n 10 weeks (A, *p*<0.05 Mantel–Cox Logrank test, n = 10) of age were infected intranasally with *emm75 S. pyogenes* (1.1×10^8 ^cfu) and sampled non-invasively through direct nasal sampling over 21 days. Male mice carried *S. pyogenes* for significantly longer than the female mice in this age group. Shedding intensity maps display the data from direct nasal samples throughout the time course from the male mice (B) and the female mice (C). Rows indicate individual mice throughout the time course, colours indicate the numbers of *S. pyogenes* recovered from direct nasal samples with red indicating the highest recorded levels of carriage (≥5000 cfu) and green indicates the lowest levels of carriage (1 cfu) and blank blocks indicate no recovery of *S. pyogenes*. Black line indicates survival based on the first loss of carriage.

To determine whether gender influenced *S. pyogenes* infection of the nasopharynx, peri pubertal (n = 10 per group, 5 per cage, 5 weeks of age) and post pubertal (n = 10 per group, 5 per cage, 10 weeks of age) male and female FVB/n mice were intranasally inoculated with *S. pyogenes.* There was no significant difference in infection duration between 5 week old males and females over a 21 day period (Figure 4, Logrank *p*>0.05), whereas 10 week old males shed *S. pyogenes* for significantly longer than 10 week old female mice (Figure 5, Logrank test *p*<0.05). However, the older males had to be housed separately to prevent intraspecific aggression and for this reason further experiments were conducted using female mice.

### Δ*covR/S* Mutation is Detrimental to Long Term *S. pyogenes* Infection of the Nasopharynx

**Figure 6:**
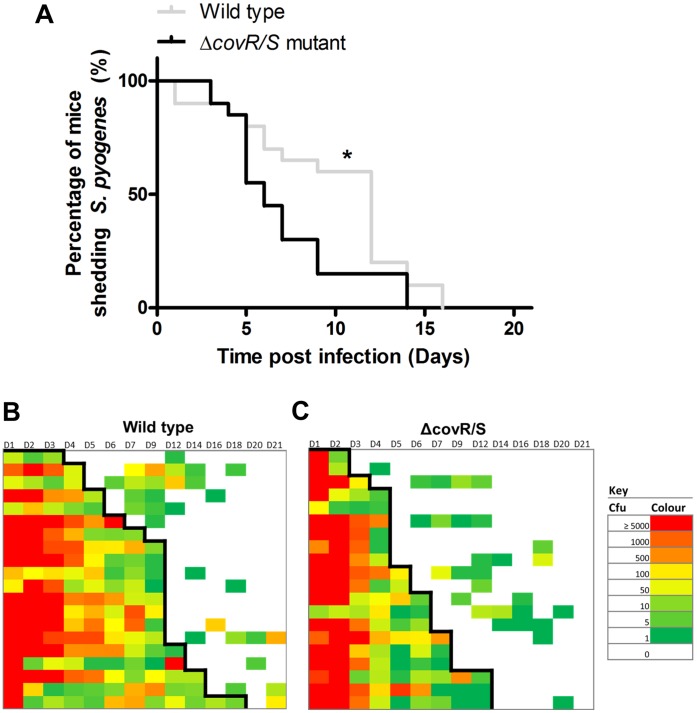
Nasopharyngeal infection is adversely affected by *covR/S* mutation. Five week old female FVB/n mice were inoculated intranasally with either *emm75 S. pyogenes*, or Δ*covR/S S. pyogenes*. To determine the duration of shedding, direct nasal samples were taken over a 21 day period. The wild type strain was shed over a significantly longer time period than the Δ*covR/S* strain (A, 1.5×10^8^ cfu per dose, n = 20 per group, Logrank Mantel Cox *p*<0.05). Shedding intensity maps display the data from direct nasal samples throughout the time course from the groups infected with the wild type *emm*75 strain (B) and the mice infected with the Δ*covR/S* strain (C). Rows indicate individual mice throughout the time course, colours indicate the numbers of *S. pyogenes* recovered from direct nasal samples with red indicating the highest recorded levels of carriage (≥5000 cfu) and green indicates the lowest levels of carriage (1 cfu) and blank blocks indicate no recovery of *S. pyogenes*. Black line indicates survival based on the first loss of carriage.

Two groups of FVB/n female mice (n = 20, 5 per cage) were infected intranasally with 5 µl of 10^8 ^cfu of *emm*75 *S. pyogenes* or an isogenic Δ*covR/S* strain and observed over 21 days. Kaplan Meier analysis of daily nasal samples demonstrated that the Δ*covR/S* strain was shed from the nasopharynx for a shorter length of time compared to its wild type counterpart. (Figure 6, Mantel Cox Logrank *p*<0.05).

**Figure 7:**
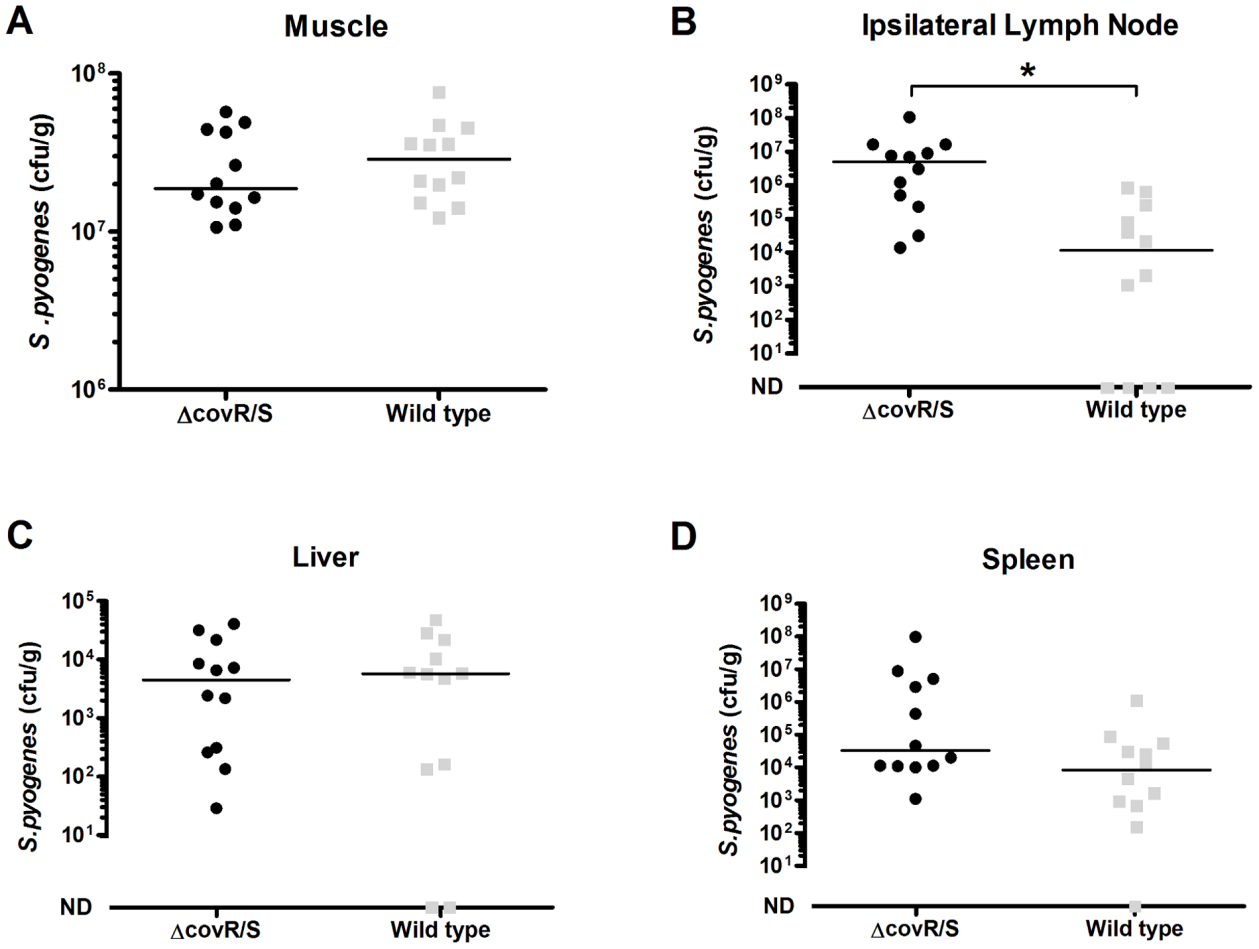
The effects of *covR/S* mutation on dissemination from an invasive intramuscular infection. Five week old FVB/n female mice (n = 12) were infected intramuscularly in the right thigh with either wild type *S. pyogenes* or an isogenic Δ*covR/S* strain (6×10^8 ^cfu per dose). After three days there was no significant difference in bacterial growth within the thigh (A, Mann Whitney U *p*>0.05), but there was a significant difference in bacterial numbers that had disseminated to the inguinal node (B, Mann Whitney U *p*<0.05). No significant difference in dissemination to the liver (C) and the spleen (D) was found between the wild type and the Δ*covR/S* strains. Median indicated by a black line.

We considered the possibility that the reduced longevity of infection reflected a general fitness defect in the Δ*covR/S* strain. However, following intramuscular infection of groups of mice with each strain, both the Δ*covR/S* strain and wild-type strain survived equally well within the thigh muscle (n = 12 per group, 6 per cage, Figure 7 A). Furthermore the Δ*covR/S* strain disseminated to the ipsilateral inguinal lymph node in greater numbers than the wild-type (Figure 7 B, *p*<0.05 Mann-Whitney), consistent with the predicted phenotype of a Δ*covR/S* strain. Streptococci were detected in the liver (Figure 7 C) and in the spleen (Figure 7 D), but these differences were not significant.

### Intranasal Administration of *S. pyogenes* Results in a Suppurative Upper Respiratory Tract Infection that Resolves Over 21 Days

**Figure 8:**
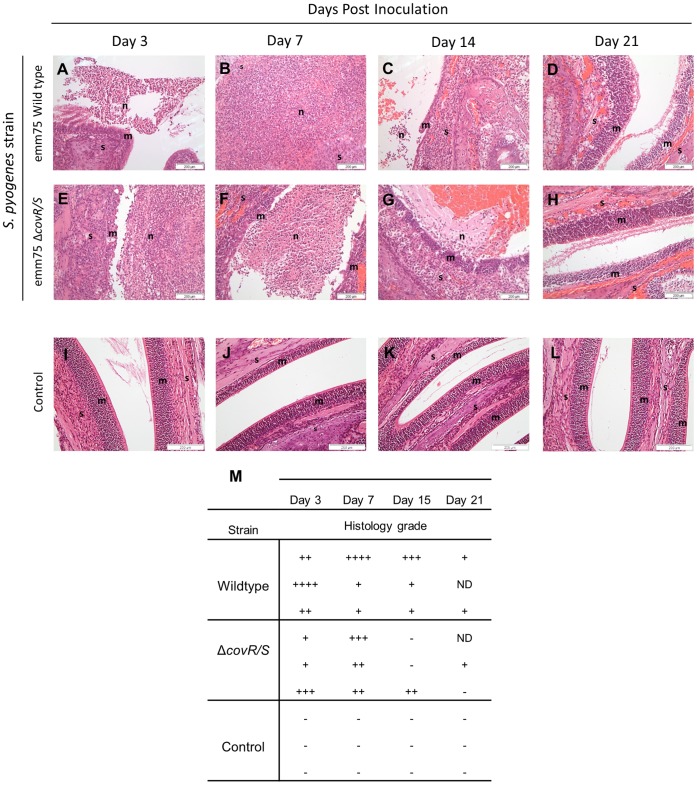
Histopathological analysis of the caudal nasal cavity during long term nasal infection. Photomicrographs demonstrating five week old female FVB/n mice infected with either the *emm75 S. pyogenes* (A–D) or the *emm75 ΔcovR/S* strain (E–H) (1.5×10^8^ cfu per dose, n = 3 per group) were taken at days 3, 7, 14 and 21 after inoculation (Haemotoxylin & Eosin staining). **n** = Neutrophilic Exudate, **m** = Nasal mucosa, **s** = Nasal stroma. Scale bar as shown. Damage to the nasal mucosa with surface neutrophilic exudate was apparent at day 3 post inoculation with both strains (A & E). The nasal epithelia of mice in both groups were widely eroded or ulcerated by day 7 (B) than those infected with the Δ*covR/S* strain (F). At day 14 the inflammation had begun to resolve in both strains (C & G). By day 21, mice in all groups had histologically normal mucosa (D & H). Control mice over the time course are shown in (I–L). Semi quantitative analysis of the histopathology was undertaken to determine the severity of infection and assigned a numerical designation for each time point (n = 3 mice per time point). − = No significant abnormality,+ = Mild,++ = Moderate,+++ = Marked,++++ = Severe and ND = Non diagnostic sections (M).

5 week old female FVB/n mice infected with *emm*75 *S. pyogenes* demonstrated inflammatory changes affecting the nasal cavity over the first week of infection (Figure 8, A–D), particularly in the caudal (ethmoturbinate) region. A moderate to marked suppurative rhinitis with complete erosion or ulceration of the nasal mucosa and neutrophilic exudate on the mucosal surface was observed. On days 3 and 7, mice occasionally demonstrated necrosis of underlying turbinate bone and extension of inflammation and infection across the cribriform plate, resulting in a localized meningoencephalitis affecting the olfactory bulbs (data not shown). However, by Day 21, the inflammatory damage to the nasal mucosa had resolved. The *S. pyogenes* Δ*covR/S* strain elicited a similar inflammatory response (Figure 8, E–H), albeit over a slightly shorter time period. Mice demonstrated mild to marked suppurative rhinitis at days 3 and 7 that resolved by day 14. Both strains of *S. pyogenes* could be recovered from cultures of the nasopharynx from mice even after the inflammation resolved (Data not shown). Semi quantitative assessment of sections from mice infected with both the wild type and Δ*covR/S emm*75 strains were undertaken in comparison with control sections from uninfected mice; this demonstrated that the observed inflammation decreased throughout the time course consistent with the lowering of the bacterial load (Figure 8 I–M).

### Transmission of *S. pyogenes* within a Mouse Cage is Dependent on the Proportion of Infected Donor Mice Present

Preliminary work showed that *S. pyogenes* shed by infected mice could lead to the infection of uninfected mice in the same cage. Transmissibility of *S. pyogenes* in the nasopharynx was formally investigated through introducing infected donor mice into cages of uninfected recipient mice (n = 8 per cage). The effect of varying the ratio of donor to recipient (D:R) mice present in a cage on transmission was evaluated.

**Figure 9:**
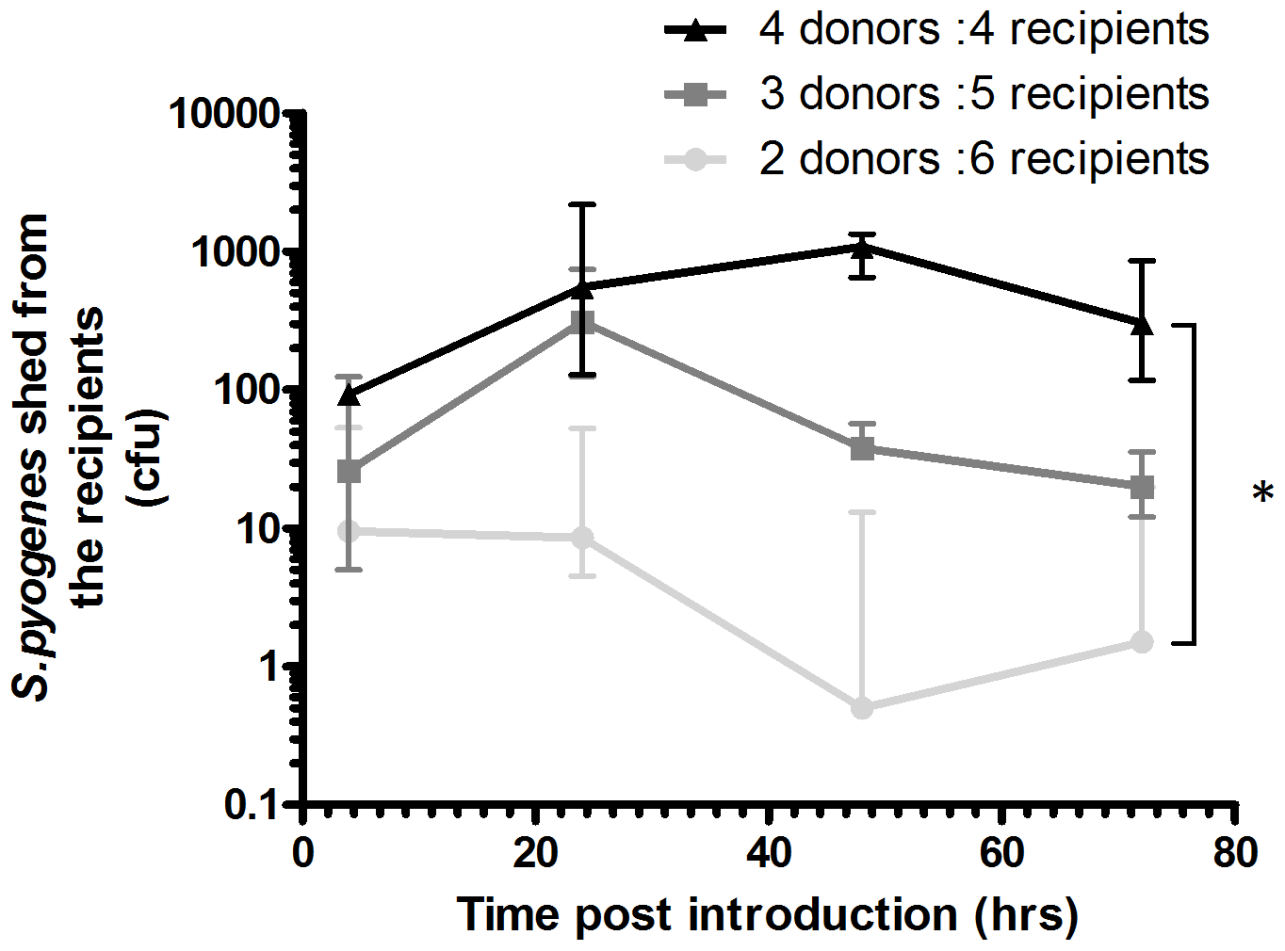
Density of infected carriers determines the burden of transmitted infection. Five week old female FVB/n donor mice infected intranasally with *S. pyogenes* (1.63×10^8 ^cfu per dose) were introduced into a cage of naïve recipient female mice. The Donor: Recipient (D:R) ratio was varied between 4∶4, 3∶5 and 2∶6 between cages. Recipient mice were sampled at 4, 24, 48, 72 and 96 hours after the infection and introduction of the donor mice to a cage. Data show counts from direct nasal sampling from recipient mice only. Donor mice had >5000 cfu recovered at all time points (not shown). The burden of transmitted infection was significantly higher in cages with a D:R ratio of 4∶4 compared with cages with a 2∶4 ratio. (AUC analysis, followed by Kruskal Wallis with Dunns Post test *p*<0.05). Lines indicate the median and the interquartile range.

Within 4 hours of donor introduction transmission occurred to all recipients. A higher D:R ratio resulted in greater counts of *S. pyogenes* cultured from recipient mice in those cages. Recipient mice in the cage with a D:R ratio of 4∶4 had significantly more bacteria in the nasopharynx compared to mice in the cage with a D:R ratio of 2∶6 (Figure 9 *p*<0.05).

### Δ*covR/S* Mutation is Detrimental to the Transmission of *S. pyogenes* in a Mouse Model

Mice were observed over the first three days of infection, when shedding of the wild-type and the Δ*covR/S* strain were shown to be similar, to determine whether a mutation in *covR/S* affects transmission.

Female FVB/n mice infected with either the wild-type or the Δ*covR/S* strains were introduced into cages at a D:R ratio of 3∶5. After inoculation, the infected mice were separated for six hours before being introduced to the recipients, to prevent passive inoculum transfer. Direct nasal samples were taken from recipient mice over three days after the introduction of the donor mice.

**Figure 10:**
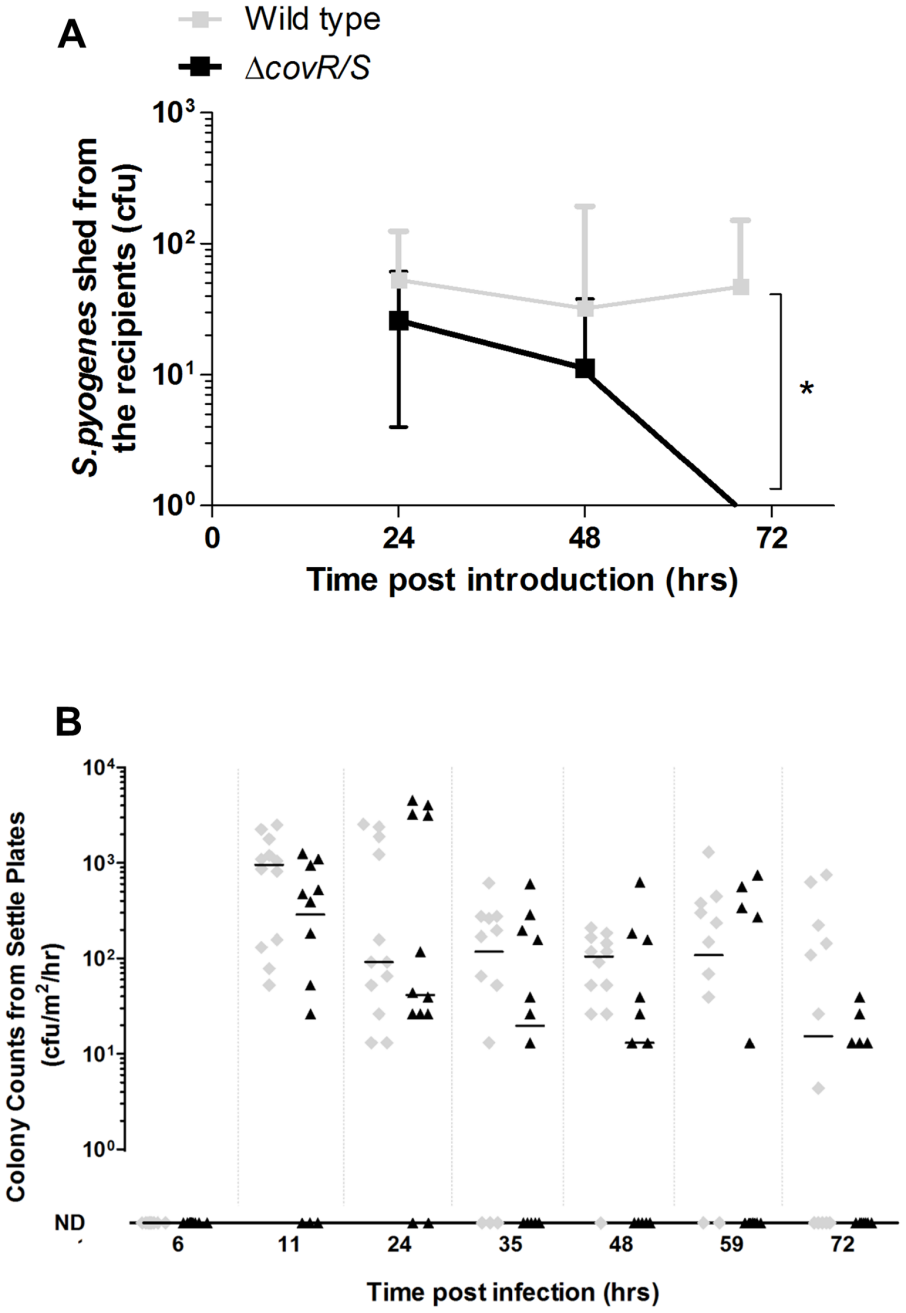
Transmission of *S.* *pyogenes* is hampered by loss of *covR/S* regulation. Naïve five week old female FVB/n recipients co-mingled at a D:R ratio of 3∶5 with female FVB/n mice infected with either the *emm*75 wild type strain or it’s isogenic Δ*covR/S* strain (5 ×10^8 ^cfu per dose) and sampled after the introduction of the donor mice. Donor mice had >5000 cfu recovered from direct nasal sampling throughout the experiment. The Δ*covR/S* strain transmitted significantly less well to recipients compared to the wild type strain (A, n = 15 recipients per group, AUC analysis, followed by Mann-Whitney U test). Line indicates median, error bars indicate interquartile range. Settle plates exposed to the air in the cages revealed no significant differences in the bacteria deposited on the surface of the plates by mice infected with the strain, or the Δ*covR/S* strain (B, n = 4 plates per cage, AUC analysis followed by Mann-Whitney U test *p*>0.05) Data is shown for individual animals with medians indicated by black line.

Recipient mice housed with donor mice carrying wild type *S. pyogenes* acquired significantly more bacteria over the time course than the recipient mice housed with donor mice carrying the Δ*covR/S* strain (Figure 10 A, AUC analysis, with Mann Whitney U *p*<0.05).

There was no statistically significant difference in the abundance of airborne *S. pyogenes* in the cages of mice infected with the wild-type compared with the cages of mice infected with the Δ*covR/S* strain. (Figure 10 B, AUC analysis with Mann Whitney U *p*>0.05).

## Discussion

To facilitate the investigation of bacterial and host factors that influence *S. pyogenes* in nasopharyngeal infection, an improved, new model of nasopharyngeal colonisation was devised, with an optimised dosing volume to avoid fulminant infections. A non-invasive method of longitudinal monitoring was developed that does not require culling of mice at multiple time points, thus reducing the numbers of animals used.

An *emm*75 strain of *S. pyogenes* was found in preliminary experiments to be carried better than other *emm* types by BALB/c mice, although previous studies have however found that the BALB/c strain is more resistant to infection than other strains [9]. A number of mouse strains were therefore tested in this study, of which the FVB/n was found to be the most susceptible to *S. pyogenes* intranasal infection. *Emm* types 1, 2, 3, 4, 6, 12, 22 and 89 were also successfully carried by FVB/n mice (data not shown), although in some cases causing a far more severe disease than the *emm*75 strain, making them unsuitable for long term infection studies.

We found that gender had an impact on susceptibility to carriage in post pubertal mice only. Data were consistent with other published work demonstrating an increase in susceptibility to infection in male mice [9], [25]. However, the intraspecific aggression expressed by males of this strain made them difficult to house in groups, and thus the older individuals were housed individually. In previous studies, housing mice singly has been demonstrated to increase immune responses [26], [27], [28], and would theoretically increase their resistance to infection.

Histological analysis during infection revealed that nasal shedding of *S. pyogenes* was associated with on-going inflammation that subsided in the second and third weeks of infection. Previous studies have demonstrated bacterial infection of the mouse NALT [6]. However this study focussed on the site of infection in the deeper nasal passages, which demonstrated a suppurative rhinitis. Studies have shown that *S. pyogenes* distributes to the ethmoid sinuses in humans during rhinosinusitis [29], [30].

The model was used to demonstrate that a functional *covR/S* is required for optimum infection and transmission from the nasopharynx. The failure of the Δ*covR/S* strain to survive in the mouse nasopharynx was not due to a consistent fitness burden, since in invasive infection, the *covR/S* strain disseminated in significantly greater numbers than the wild type bacteria to the inguinal lymph node.

During preliminary experiments, we became aware that individual mice occasionally became re-infected after clearing the initial infection. To address transmission, and the factors that may influence this, co-mingling experiments were conducted. These demonstrated that transmission occurred as early as 4 hours post introduction, and the numbers of bacteria recovered from the nares of the recipients increased as the number of infected donor mice in the cage was increased. Importantly, such transmission events may not necessarily constitute a productive infection, as *S. pyogenes* did not reach the same abundance in recipient mice as observed in donor mice.

Co-mingling was then used to examine the impact of *covR/S* on transmission. Despite the fact that the infection burden (as measured by direct nasal sampling) was similar between the two donor groups in the first 72 h, recipients housed with donor mice carrying the Δ*covR/S* were demonstrated to have a shorter shedding duration than the recipient mice housed with the donors carrying the wild type strain.

Furthermore, despite some differences between the experimental groups, settle plates placed in each cage revealed no significant difference in aerosolization between the two strains. This suggests that the impairment of the *covR/S* primarily affects the survival of *S. pyogenes* in the nasopharynx after the initial transmission event has taken place.

There is thus a fitness cost conferred by *covR/S* mutation specific to the nasopharynx that may explain why such bacteria have not become prevalent in community *S. pyogenes* pharyngitis despite being advantageous in invasive infection.

The model described represents a refinement of previous systems to study upper respiratory tract infection by *S. pyogenes*; the model is non-invasive and allows longitudinal monitoring of bacterial infection, using the same mice throughout the study. Such a model will facilitate research which might otherwise require prohibitively large numbers of animals and could be of importance in future evaluation of vaccines, antimicrobials, as well as the factors that influence transmission.
