# Correction: Inactivation of the CovR/S Virulence Regulator Impairs Infection in an Improved Murine Model of *Streptococcus pyogenes* Naso-Pharyngeal Infection

**DOI:** 10.1371/annotation/1144e132-9e69-47bb-8e65-1414dbb01db7

**Published:** 2013-09-09

**Authors:** Faraz M. Alam, Claire E. Turner, Ken Smith, Siouxsie Wiles, Shiranee Sriskandan

There were errors in the Funding statement. The first sentence of the Funding Statement should read: "This work was supported by the Biotechnology and Biological Sciences Research Council via the Centre for Integrative Mammalian Physiology and Pharmacology and the National Centre for Replacement, Refinement and Reduction of Animals in Research."

In addition, in the Author Contributions statement the name of the second author was incorrectly abbreviated and the fourth author was incorrectly omitted from the list of those authors who conceived and designed the experiment. The Author Contributions statement should read: "Conceived and designed the experiments: FA, SW, SS. Performed the experiments: FA. Analyzed the data: FA, KS. Contributed reagents/materials/analysis tools: CET, SW, SS. Wrote the paper: FA, CET, KS, SW, SS." 

